# Removal of a Necrosed Lower Maxilla, with Preservation of the Periosteum and the Reproduction of New Bone

**Published:** 1863-11

**Authors:** 


Removal of a Necrosed Lower Maxilla, with Preservation of the Periosteum and the Reproduction of New Bone.—M. Rizzoli, of Bologna, has submitted to the Surgical Society of Paris a case of necrosis of the lower jaw, from the fumes of phosphorus, in a man aged fifty-six years, in which the sequestra were removed through the mouth. M. Rizzoli made incisions on either side of the gums, scraped the thickened periosteum with a spatula from the dead bone, and removed the latter piecemeal. The preserved periosteum generated new bone in the place of the portions taken away, which comprised the body and part of the ramus on each side. It was, however, soon found that the upper part of the ramus and the condyle were also diseased; these portions of bone were also removed through the mouth with the same precautions, and the periosteum again acted in the same way. Eventually the man was able to use his jaw, and masticate, though deprived of teeth. M. Forget, who reported on the case, observed very justly that there was nothing new in this action of the periosteum in necrosis of bone, surgeons having long acted upon this periosteal property in such cases. M. Flourens has pointedly said, "Take away the bone, preserve the periosteum, and the preserved periosteum will restore the bone," but this applies less to the cases of necrosis of bone than to cases of experiments on animals and operations performed on healthy bone and periosteum. And even in these cases it should be remembered that osseous substance is reproduced, but not the actual bone as it existed before the resection.—*Lancet.*

